# High-Strength Ductility Joining of Multicomponent Alloy to 304 Stainless Steel Using Laser Welding Technique

**DOI:** 10.3390/ma16062374

**Published:** 2023-03-16

**Authors:** Junjie Wang, Fei Peng, Li Zhou, Yajun Luo, Weidong Zhang, Zhenggang Wu

**Affiliations:** 1 College of Materials Science and Engineering, Hunan University, Changsha 410082, China; 2 Hunan Engineering Research Center of New Energy Vehicle Lightweight, Hunan Institute of Engineering, Xiangtan 411104, China

**Keywords:** multicomponent alloy, austenitic stainless steel, dissimilar laser welding, microstructure, mechanical properties

## Abstract

In this work, a series of multicomponent alloys (CoCrFeNi, CoCrNi, and CoNiV) were laser welded with 304 stainless steel (304ss), and detailed comparisons on microstructural characteristics and mechanical properties were conducted for dissimilar laser welded joints. It is revealed that all of the dissimilar laser welded samples possessed defect-free joints and the corresponding fusion zone consisting of fcc single-phase showed homogeneous element distribution accompanied by a narrow element gradient in the vicinity of the fusion zone boundary. After laser welding with identical welding parameters, equiaxed grain was observed on the side of multicomponent alloy, while coarse columnar grain was obtained on the side of 304ss. Especially, the columnar grains of the fusion zone on the side of 304ss disclosed preferential <001> growth direction in the CoCrFeNi/304ss and CoCrNi/304ss welded joints. Furthermore, all of the dissimilar laser welded joints were fractured in the fusion zone, attributing to the drastic loss of strength in the fusion zone with coarsened grain. It is worth noting that a special lamellar structure that merged by dimples was found in the fracture surface of the CoNiV/304ss joint, closely related to the existence of the V-enriched region. Finally, a high strength–ductile synergy can be achieved by laser welding CoNiV alloy to 304ss, which showed a yield strength of 338 MPa, ultimate tensile strength of 686 MPa, and total elongation of 28.9%. These excellent mechanical properties prevailed in the potential of a CoNiV/304ss laser welded joint to be applied as a structural material.

## 1. Introduction

In recent years, high entropy alloy (HEA) has attracted extensive attention due to its excellent mechanical properties [1,2,3,4], corrosion resistance [5], and fatigue resistance [6]. Compared with a traditional alloy with one or two principal elements, HEAs generally contain at least four principal elements on the basis of the concept of multicomponent alloy (MCA) [7,8]. With the further development of multicomponent alloys, the medium-entropy alloys (MEAs) are found to present comparable or superior mechanical performance to those of HEAs. For example, the CoCrNi medium-entropy alloy presents a tensile strength of 884 MPa, total elongation of 73%, and fracture toughness of 208 MPa m^1/2^ at room temperature [3], which are slightly superior to the reported results of representative HEA. Moreover, a high-strength CoNiV medium-entropy alloy can be obtained with a yield strength of ~1 GPa, tensile strength exceeding 1200 MPa, and fine total elongation of 38%, showing great potential for structural applications [9].

Although the MCAs, including HEAs and MEAs, possess excellent mechanical performance, it is reliable to deduce that these expensive alloys with a relatively higher density were more suitable to be applied in the key section of structural part or partially replacing traditional alloy. Hence, the joining technique, especially the welding technique, of multicomponent alloy and traditional metal is critical to be tackled. It has been reported that the similar welding of HEAs (such as CoCrFeNiMn) and MEAs (such as CoCrNi) both demonstrated excellent mechanical properties [10,11,12]. However, the dissimilar welding between multiple-component alloy and traditional alloy (such as austenitic stainless steel) is very complicated due to the existence of a nonequilibrium solidification environment in the fusion zone (FZ) and the associated formation of harmful phases [13] to increase the brittleness of the welded joint. Up to now, only a few articles were reported about the dissimilar welding of multiple-component alloy with austenitic stainless steel [13,14,15,16,17] Oliveira et al. [17] investigated the dissimilar laser welding of a CoCrFeMnNi high entropy alloy to 316 stainless steel and found that a new solid solution formed in the welded joint area. Concurrently, the C element in 316 stainless steel can effectively enhance the hardness of welded joints. Yan et al. [15] researched the laser welded joint of CoCrNi and 301 stainless steel and achieved a synergistic improvement of strength and ductility, exceeding all other existing results of similar and dissimilar HEA/MEA joints. However, among the reported works, due to the discrepancy in welding equipment, welding parameters, and the state of base materials, it is very difficult to directly optimize the species of multicomponent alloy to obtain a dissimilar laser welded MCA/304ss joint with a combination of high strength and excellent ductility.

In our work, three representative MCAs (CoCrFeNi, CoCrNi, and CoNiV) that ranged from HEA to MEA were selected and further laser welded with 304 stainless steel under identical experimental conditions. The characteristics of microstructure and element distribution of welded joints were compared to analyze the welding process. Furthermore, a detailed comparison of joint mechanical performance was conducted to determine the optimized MCA that was laser welded with 304 stainless steel.

## 2. Materials and Methods

**Figure 1:**
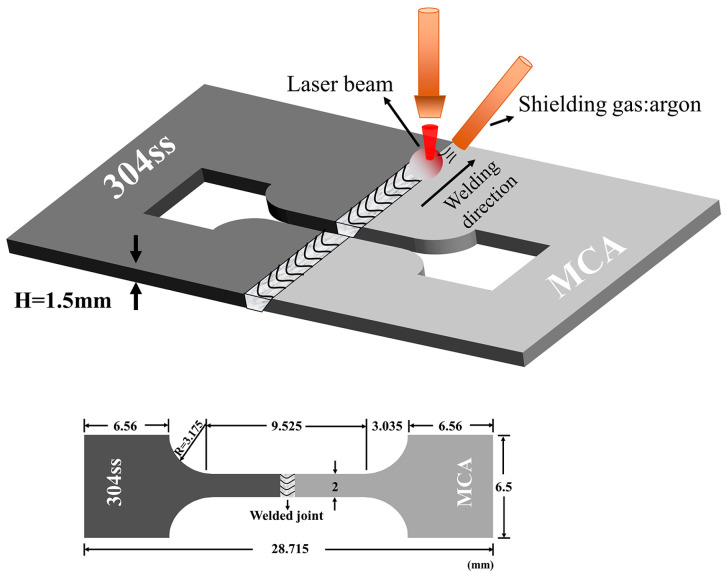
Schematic diagrams of the laser welding process and preparation of the tensile sample.

The multicomponent alloys (CoCrFeNi, CoCrNi, and CoNiV) used in this paper were produced from pure Co, Cr, Fe, Ni, and V particles (purity ≥ 99.9%) by vacuum arc melting suction casting furnace. Subsequently, the ingots of multicomponent alloys were cold rolled into 1.5 mm and then annealed at 900 °C for 1 h followed by water quenching to ambient temperature. The commercial 304 stainless steel (hereafter referred to as 304ss) with a thickness of 1.5 mm was also prepared as a base metal. The chemical compositions of all base materials are listed in Table 1. Before welding, all sheets of base metals were cut into pieces with dimensions of 25 mm × 25 mm × 1.5 mm (thickness). Subsequently, the surfaces of the pieces to be joined were ground with silicon carbide paper and cleaned ultrasonically with alcohol. The welding experiments were carried out using a continuous fiber laser beam with a power of 2100 W and a speed of 200 mm/min. It should be noted that the laser beam was focused on the upper surface of the joining area throughout the welding process. The welding process was graphically illustrated in Figure 1.

The cross section of the as-welded sample was machined to reveal the characteristics of the welded joint. Subsequently, the microstructure and elemental distribution of the joints were characterized by scanning electron microscopy (SEM) combined with energy dispersive spectroscopy (EDS). The grain size of the FCC phase in this paper was measured by the line-intercept method on the basis of several SEM micrographs, among which more than 100 grains were measured. The constituent phases of the base metal were determined by X-ray diffractometry (XRD) with Cu Kα radiation and a 2θ range of 40~80° (step size: 4°/min). In addition, the micro-XRD technique with a detected diameter of ~100 μm was applied to characterize the microstructure at the center of the fusion zone. The phase distribution and orientation characteristics of the joint were characterized by the electron back-scattered diffraction (EBSD) technique (tilt angle: 70°; step size: 3.5 µm) and postprocessed by channel 5 software. All of the aforementioned samples were well ground and vibration polished.

The Vickers microhardness mapping of the welded joint was obtained with a load of 200 g and an indentation spacing of 50 μm. The microhardness, with about 2100 points, was measured throughout the fusion zone and then the data were postprocessed by a grid data function in python 3.0 to generate a heatmap. To evaluate the mechanical properties of the joint, dog-shaped specimens with a gauge length of 9 mm were prepared and applied for uniaxial tensile tests with a strain rate of 10^−3^ s^−1^. Note that, three samples were conducted for identical welding conditions.

## 3. Results and Discussion

### 3.1. Microstructural Evolution during the Welding Process

#### 3.1.1. Characterization of Basic Microstructure before Welding and Constituent Phases of Base Metals

**Figure 2:**
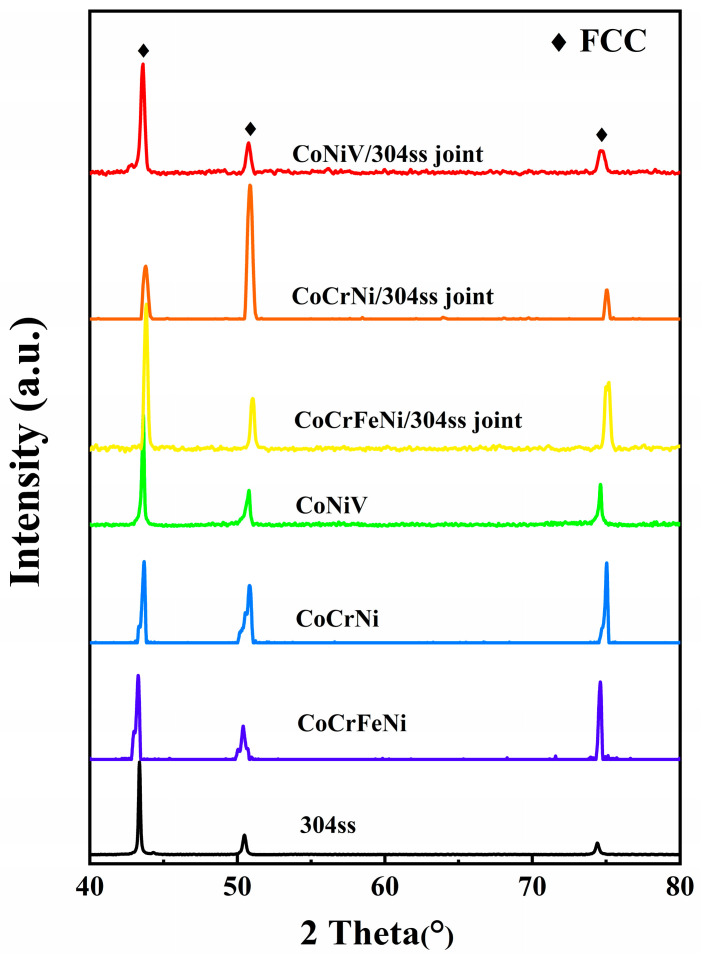
XRD patterns of base materials before welding and the corresponding fusion zone of the welded joint.

**Figure 3:**
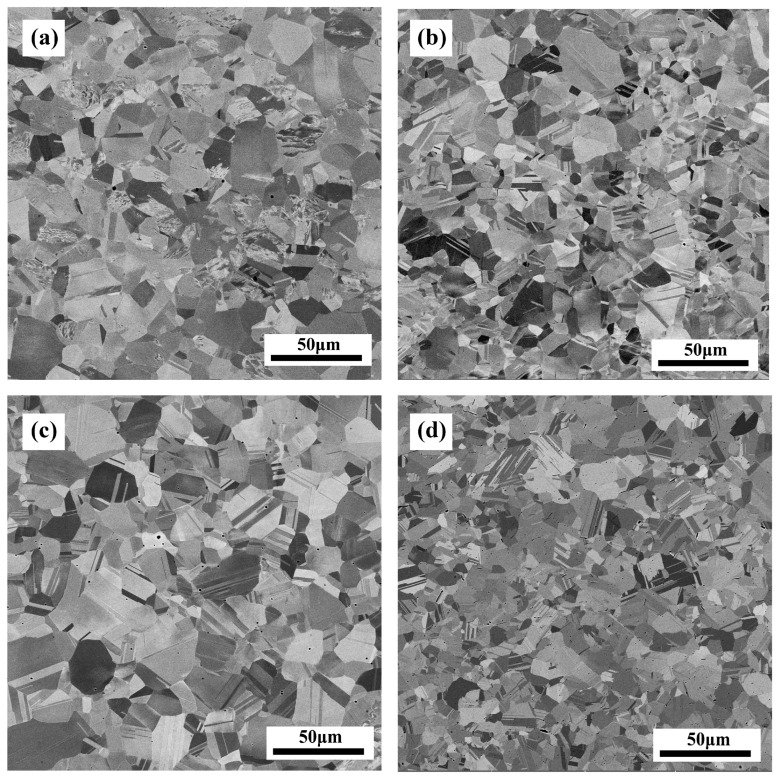
SEM image of base material before welding. (**a**) 304ss; (**b**) CoCrFeNi; (**c**) CoCrNi; (**d**) CoNiV.

In order to compare the microstructural characteristics of base materials before welding, the constituent phases of base materials before welding were determined by XRD, as shown in Figure 2. Concurrently, the basic microstructures of the multicomponent alloy and 304ss before welding were characterized by SEM (back-scattered mode), and the corresponding results are shown in Figure 3. It is clear that all of the base metals before welding are comprised of a single phase with a face-centered cubic (FCC) structure, while the discrepancy in the XRD peak position is ascribed to a different chemical composition (Figure 2). Furthermore, it is well-known that the distribution of XRD peak intensity was closely related to the characteristics of texture. In the case of basic microstructure before welding, this distribution was a result of annealing texture, which was relevant to the nature of the alloy and the characteristics of the as-rolled microstructure. As to the fusion zone after welding, the texture was mainly influenced by the solidification process. Hence, it is reliable to deduce that the abnormal XRD pattern of the CoCrNi/304ss joint may be corresponded to a special solidification process. In addition, the microstructure of base metals before welding possesses an equiaxed grain, which is typically fully recrystallized microstructure. It can also be found that an abundant number of annealing twins are observed in all the base metals, which is closely related to the low stacking fault energy of 304ss and MCAs [18,19]. Furthermore, the corresponding average grain size of base metals was measured by the linear intercept method and the results are summarized in Table 1. It can be found that the initial grain sizes of base metals are ranged from 12 μm to 16 μm, which can be basically regarded as at the same level.

#### 3.1.2. Analysis of Microstructural Characteristics and Element Distribution after Welding

The constituent phases of the fusion zone (FZ) after welding are characterized by the micro-XRD technique and the results are presented in Figure 2. It is revealed that the fusion zone consists of an FCC single-phase, which demonstrates that no new phase or intermetallic compound is formed in the weld joint during welding process.

**Figure 4:**
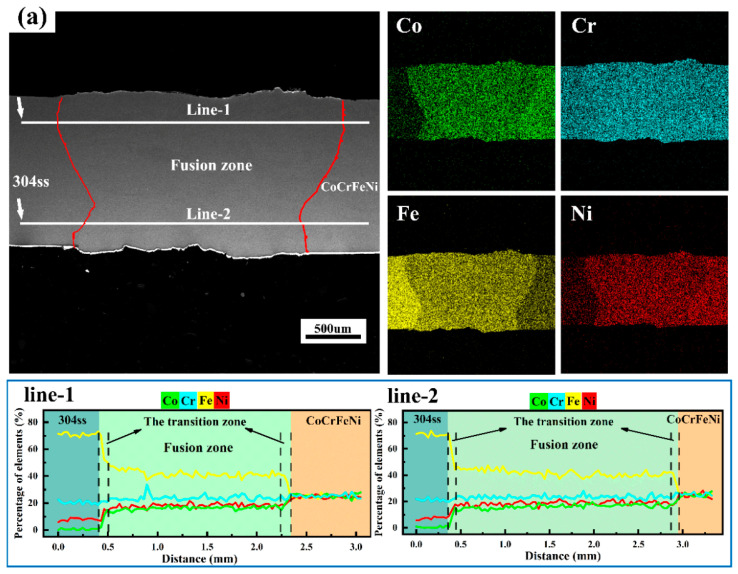

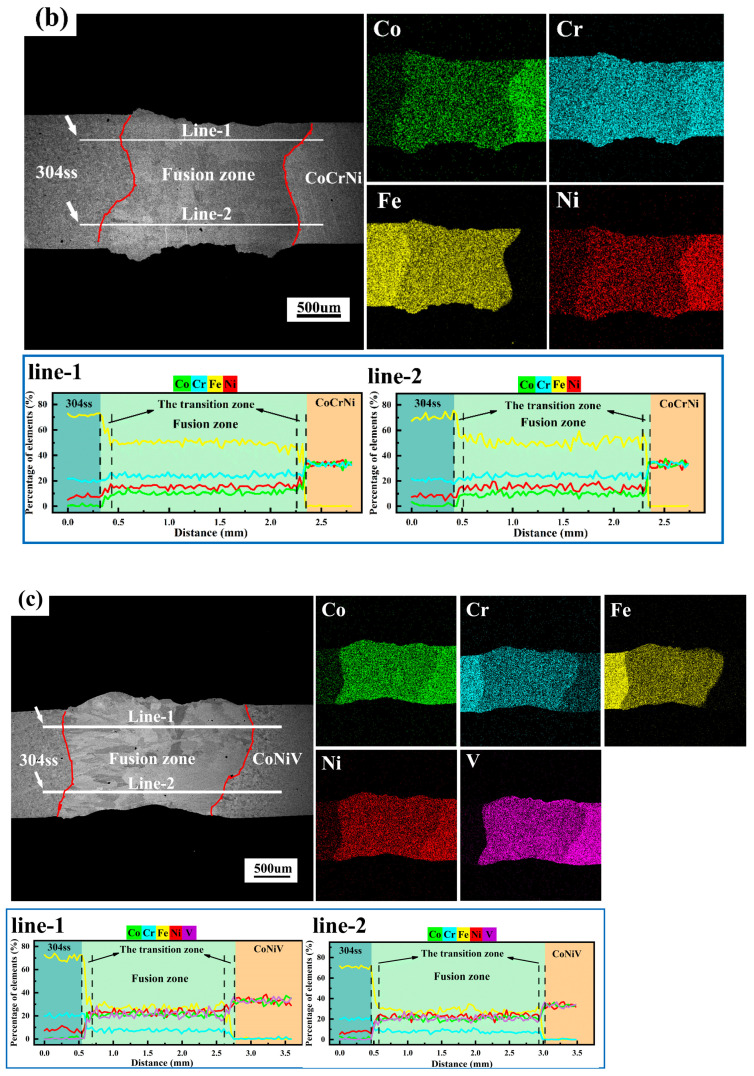
Overview of the dissimilar laser welding joint and corresponding EDS results. (**a**) CoCrFeNi/304ss welded joint; (**b**) CoCrNi/304ss welded joint; (**c**) CoNiV/304ss welded joint.

In order to reveal the evolutions of microstructure and element during the welding process, the cross sections of dissimilar laser welded joints were characterized in detail. The overview image of the welded joint and the corresponding element distribution is depicted in Figure 4. It can be observed that all dissimilar welded joints present fine weld geometry and weld penetration throughout the base material without any volumetric or macro welding defects. This result indicates that the application of laser welding to join 304ss and MCA (CoCrFeNi, CoCrNi, or CoNiV) presents good weldability.

Furthermore, the geometry of a dissimilar welding joint was closely related to the species of MCA, which shows an approximately symmetrical waist-type structure in the CoCrFeNi/304ss joint and the CoCrNi/304ss joint, while the CoNiV/304ss welded joint presents an asymmetric shape consisting of a similar waist-type structure on the side of 304ss and a funnel shape on the side of CoNiV. It is well-known that the waist-type joint is a typical feature of laser penetration welding [20]. During this welding process, the heat input of the laser is enough to rapidly heat base material to be vaporized accompanied by the formation of plasma. Meanwhile, a keyhole forms in the center of the weld pool due to the existence of steam pressure [21]. As the laser welding process proceeded, the keyhole further penetrated throughout the entire welded joint. At present, the convection of the upper and lower surfaces of the welding pool was established, resulting in the increase of width for both the upper and lower surfaces of the welding pool, and thus a waist-type welded joint was obtained [22]. As to the CoNiV/304ss welded joint, the asymmetric shape can be attributed to the obvious discrepancies in thermal physical properties such as conductivity and the ability of laser absorption between CoNiV and 304ss. When CoNiV and 304ss were both irradiated with identical laser energy, it can be deduced that the CoNiV possesses a relatively smaller melting efficiency and hence the laser energy absorbed by the joint adjacent to the side of CoNiV was insufficient to form convection under the molten pool. This result significantly affects the boundary of the fusion zone [23], resulting in the asymmetric shape of the CoNiV/304ss welded joint.

According to the elemental distribution of Figure 4, it can be found that all of the dissimilar welding joints can be divided into three regions, i.e., 304ss, FZ, and MCA. It can be found that there was no existence of a precipitate or second phase, consistent with the results of XRD (Figure 2). Meanwhile, the element distribution among FZ was homogeneous, which can be mainly attributed to the high solid solution of all elements (Fe, Ni, Co, Cr, and V) in liquid metal and dramatic internal convection among the welded pool during laser welding [24]. Moreover, the EDS line scanning results demonstrate that the homogenous distribution of elements along the cross-sectional direction is analogous in both the upper and lower surfaces of the molten pool, whilst a narrow transition region with gradient distribution of all elements was observed in the vicinity of the FZ boundary.

Moreover, it is clear that the formation of carbides, such as M_23_C_6_ and M_7_C_3_, was not found in all as-welded samples. Due to the application of MAC without C addition, the C content of the fusion zone further decreased as a result of mixing, which will prolong the incubation time of carbide and hence retarded carbide formation. In addition, the laser welding method applied in our paper possesses the advantages of concentrated energy, fast solidification speed, and so on, which hardly satisfies the necessary incubation time of carbide during the cooling process. Hence, the C atoms tend to be dissolved into the fusion zone in the form of interstitial atoms. Similarly, Oliveira et al. [17] investigated the laser welding process of 316 stainless steel and CoCrFeNiMn, and they found that the C atoms were dissolved in the fusion zone without the formation of carbide, which can make a solid-solution strengthening effect to improve the mechanical performance of the molten zone.

**Figure 5:**
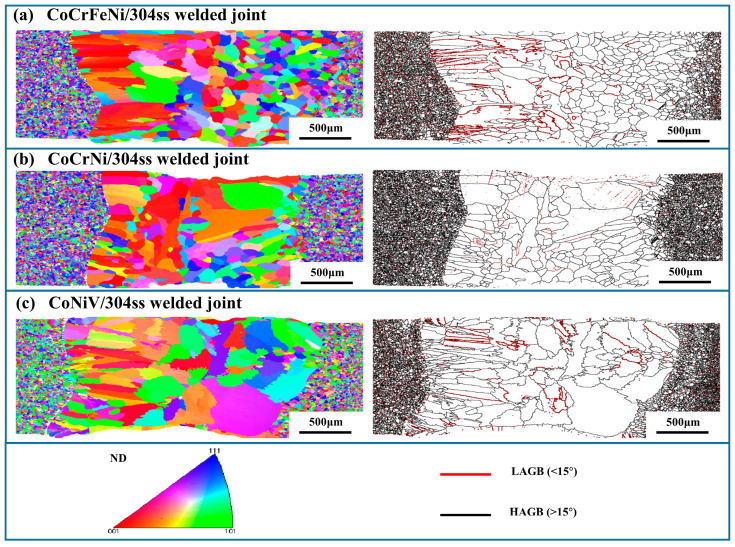
IPF images and grain boundary diagrams of different welded joints obtained by EBSD. (**a**) CoCrFeNi/304ss welded joint; (**b**) CoCrNi/304ss welded joint; (**c**) CoNiV/304ss welded joint. Note that, LAGB and HAGB indicate low-angle grain boundary and high-angle grain boundary, respectively.

In order to further analyze the microstructural characteristics of the dissimilar laser welding joint, the inverse pole figure (IPF), and the distribution of the grain boundary of welded joints were detected by EBSD, as shown in Figure 5. It is clear that the microstructural differences between FZ and the remaining areas of the welded joint significantly depended on the species of MCA. As for the fusion zones of the CoCrFeNi/304ss and CoCrNi/304ss joints (Figure 5a,b), abundant coarse columnar grains with a preferential <001> growth direction were observed on the side of 304ss. This preferential growth direction is typical evidence of the competitive growth phenomenon [25], among which the direction <100> of FCC metal with maximal growth rate was consistent with the direction showing maximal temperature gradient. As a result, the <100> direction of grains in the FZ rapidly grew up to show a columnar morphology. It should also be noted that a larger temperature gradient was formed on the side of 304ss due to the relatively smaller conductivity of 304ss, resulting in the occurrence of grains with more distinct columnar morphology in the FZ adjacent to the 304ss base metal. However, due to the discrepancies in molten pool size and temperature gradient, the growth rate of diverse grain orientations may exceed that of <100> direction [26], which is the main reason for CoNiV/304ss welded joints hardly show preferential grain growth of <100> direction. Moreover, as for the welded joint of CoNiV/304ss, the significant differences in chemical composition and crystal structure between the molten pool and the CoNiV played a vital role in reducing the preferential (100) direction grains on the side of 304ss and restricting the columnar crystal competitive growth.

As a comparison, the grain in the FZ near the side of MCA shows equiaxed or approximately equiaxed morphology in all welded joints (Figure 5a–c), except a few of the coarse columnar grains, were located on the upper and lower surfaces of the CoCrNi and CoNiV sides. Particularly, the equiaxed grain on the side of CoCrFeNi shows a much smaller size as compared to that on the side of other MCAs, which was controlled by the discrepancy in chemical composition and crystal structure between the molten pool and the base material [24]. During the solidification process, the thermal conductivity of MCAs is lower than that of 304ss [27]. As a result, the 304ss can act as a gap in heat output of the molten pool [28], causing a decrease in the temperature gradient on the side of the MCA. Accordingly, abundant nucleation sites were obtained near the side of MCA and hence the grain grew up rapidly. In general, the undercooling gradually increases from the fusion line to the center of the molten pool [29], which further promotes the growth of equiaxed grains in the molten pool adjacent to MCA.

### 3.2. Mechanical Properties and Fracture Mechanism of Welded Joints

#### 3.2.1. Comparison of Microhardness Distribution in As-Welded Samples

**Figure 6:**
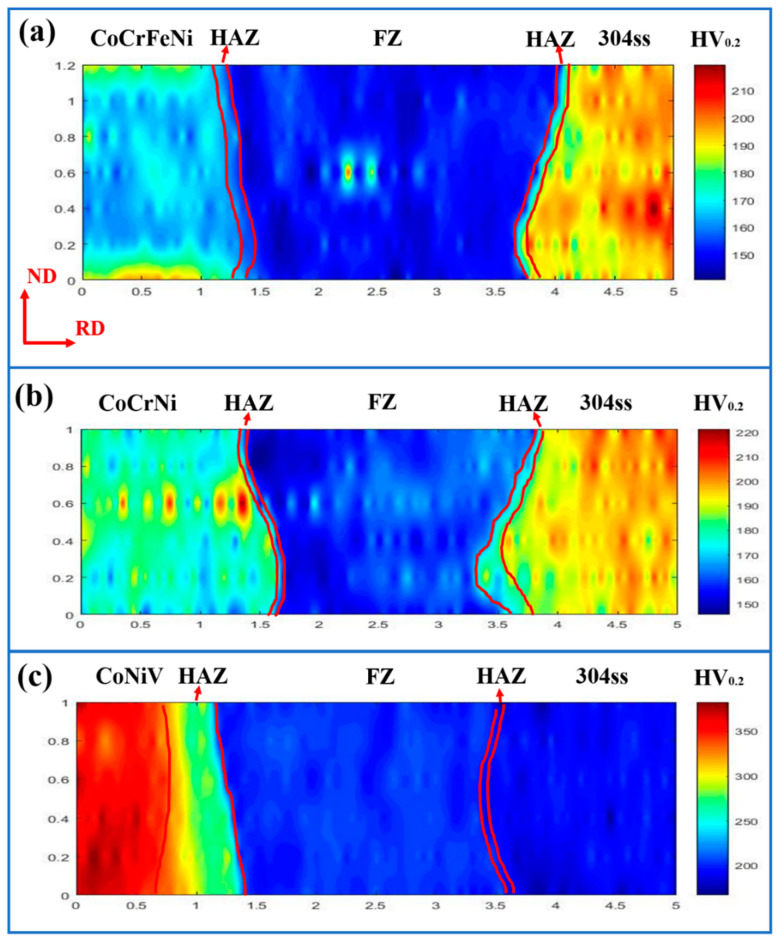
Microhardness distribution of welded joints. (**a**) CoCrFeNi/304ss welded joint; (**b**) CoCrNi/304ss welded joint; (**c**) CoNiV/304ss welded joint.

The microhardness distribution of the welded joint was measured by mapping mode and the results were described by heatmaps as shown in Figure 6. The average hardness of base materials and FZ in the welded joint were also summarized in Table 2. As for CoCrFeNi/304ss and CoCrNi/304ss welded joints, it can be found that the hardness of CoCrFeNi (166 HV) and CoCrNi (179 HV) was lower than that of 304ss (197 HV), while the fusion zones possess the lowest hardness with approximate values of 152 HV and 158 HV, respectively. This distinct reduction in the hardness of the FZ was mainly associated with the formation of coarse grains during the welding solidification process [30]. As a comparison, the CoNiV base metal shows the largest hardness (353 HV) in CoNiV/304ss welded joints. Concurrently, the hardness of FZ (203 HV) presents an analogous value as that of 304ss (197 HV), although a much coarser grain was obtained in the FZ (Figure 3a). This phenomenon can be deduced as a result of the significant solution-strengthening effect of the V element [31].

Moreover, it is well known that the concentrated heat input and associated confined heat affected zone (HAZ) are the representative advantages of laser welding [32], which is obvious in CoCrFeNi/304ss and CoCrNi/304ss welded joints (Figure 6a,b). It can also be found that the hardness of confined HAZ was reduced as compared to the adjacent base material, due to the existence of partial-recrystallized grain suffered during the welding thermal cycle. However, in the case of the CoNiV/304ss welded joint, an expanded HAZ with reduced hardness was observed adjacent to the CoNiV base metal (Figure 6c), whilst the width of HAZ on the side of 304ss was still similar to that of the other welded joints. These expanded HAZ were also observed in the laser dissimilar welding joint of CoCrFeNiMn and dual-phase steel [24].

#### 3.2.2. Mechanical Behavior of Welded Joints in Macroscale

**Figure 7:**
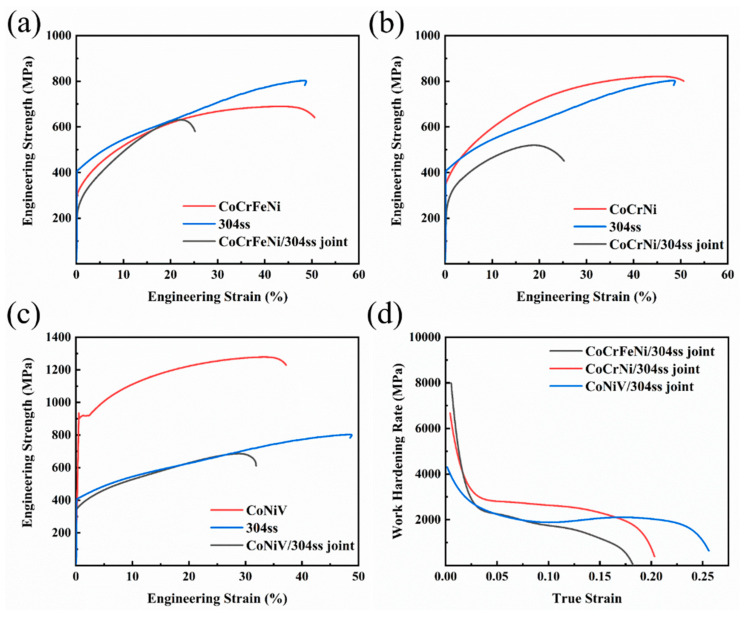
Mechanical behaviors of laser dissimilar welded joints. (**a**–**c**) Engineering stress–strain curves; (**d**) work hardening rate.

The engineering stress–strain curves of base materials and as-welded samples are shown in Figure 7a–c, and the corresponding representative mechanical properties are summarized in Table 3. In comparison with the base materials, it can be found that all of the welded joints demonstrate a significant decrease in both strength and ductility. As to the welded joint of CoCrFeNi and 304ss, the yield strength (YS) and ultimate tensile strength (UTS) can be respectively achieved as 237 MPa and 510 MPa, while the total elongation (TEL) was 22.6%. In comparison with the CoCrFeNi alloy, the CoCrNi possesses improved strength and ductility. Accordingly, the laser welded CoCrNi/304ss joint presents optimized mechanical properties compared to those of the CoCrFeNi/304ss joint, i.e., improved strength and slightly deteriorated ductility were obtained. As for the CoNiV alloy, the values of YS (906 MPa) and UTS (1279MPa) were the highest among all of the base materials, while fine ductility with the smallest TEL value of 33.2% was obtained. Correspondingly, the laser welded joints of CoNiV/304ss possessed the optimized mechanical properties in all welded joints, i.e., the strength and ductility were improved synergistically. The corresponding highest YS and UTS were, respectively, 338 MPa and 686 MPa, whilst the optimized TEL was achieved as 28.9%.

Figure 7d depicts the work-hardening rate of different welded joints. It is revealed that the work-hardening ability of the CoCrNi/304ss welded joint is superior to that of the CoCrFeNi/304ss welded joint throughout the tensile process, while the variation trend is similar in these two welded joints, i.e., decreased rapidly at the onset of plastic deformation followed by a sluggish reduction during the remaining process. In comparison with the CoCrFeNi/304ss welded joint, the application of CoNiV instead of CoCrFeNi deteriorates the ability of work hardening during initial plastic deformation, while nearly identical and further superior at the latter part of plastic deformation.

#### 3.2.3. Analysis of Fracture Feature in As-Welded Samples

**Figure 8:**
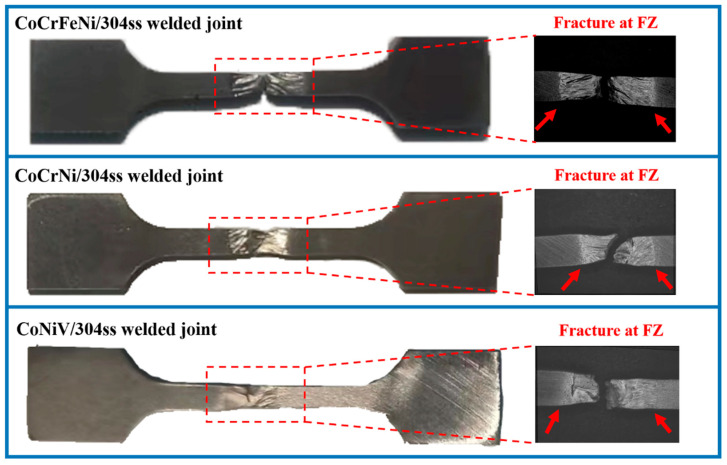
Optical images of the as-welded sample after tensile test. Note that, the arrows indicate the boundaries of severe plastic deformation.

In order to determine the fracture mode of as-welded samples, the laser welded joints after a tensile test were observed in the macroscale, as shown in Figure 8. It is obvious that all of the samples were fractured in the FZ, which indicated that the failure of the laser welded joints estimated in this paper was mainly attributed to the drastic loss of strength in the FZ (Figure 6), which mainly originated from the coarsened grains of FZ [33]. In other words, the softened FZ undertook more plastic deformation than other regions during the tensile process [34] and hence necking and fracture were concentrated in the FZ. It is worth noting that there is a vivid boundary between a severely deformed fusion zone and uniformly deformed base material.

**Figure 9:**
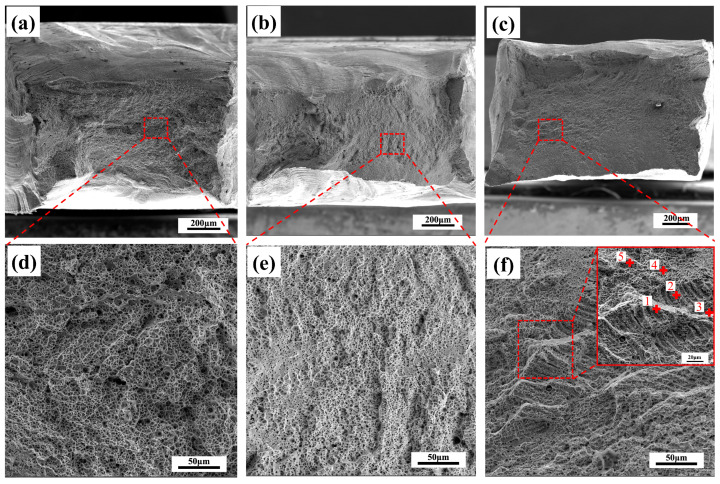
Fracture morphology of as-welded samples. (**a**,**d**) CoCrFeNi/304ss welded joint; (**b**,**e**) CoCrNi/304ss welded joint; (**c**,**f**) CoNiV/304ss welded joint. Note that the inset in (**f**) discloses the EDS positions with numbers of 1~5.

The fractography of as-welded samples is displayed in Figure 9. It can be clearly observed that the fracture surfaces of the CoCrFeNi/304ss and CoCrNi/304ss welded joints were filled with equiaxed dimples, which is the typical feature of ductile fracture. In addition, the dimple depth of the CoCrFeNi/304ss welded joint is much larger, indicating an improved toughness as compared to that of the CoCrNi/304ss welded joint [35], which is consistent with the superior ability of plastic deformation as mentioned in Figure 7d. As a comparison, the failure mode of the CoNiV/304ss welded joint can also be determined as ductile fracture filled with dimples, while two different kinds of dimples were observed. As shown in Figure 9f, except for the existence of equiaxed dimples that are identical to other welded joints, some equiaxed dimples were aggregated to present lamellar characteristics with a vivid and strictly parallel boundary. This lamellar structure and the abundant steps observed in Figure 9f were deduced to be the feature of displacive deformation, which is consistent with the aforementioned discussion that the FZ of the CoNiV/304ss welded joint withstood more plastic deformation.

Furthermore, some representative positions of Figure 9f were analyzed by the EDS technique, and the results are shown in Table 4. It is depicted that the lamellar structure was V-enriched, while the other region with equiaxed dimples shows identical chemical composition to that of the entire FZ. This result may be ascribed to the elemental segregation deriving from the formation of dendrites during welding process [26,36]. It has been reported that the enrichment of V can play a significant solid solution-strengthening effect [37]. As a result, the V-enriched region deformed at the latter part of the tensile test, which means that some features of displacive deformation, such as slip band, can be retained to influence the formation and coalescence of a microvoid, resulting in the occurrence of lamellar structure in the fracture surface. It is worth noting that the work hardening rate of the CoNiV/304ss welded joint presents a diverse variation as compared to those of other joints (Figure 7d), which was reliable to be closely associated with the existence of the V-enriched region.

### 3.3. Comparison and Evaluation of Strength-Ductility Synergy of Welded Joints

**Figure 10:**
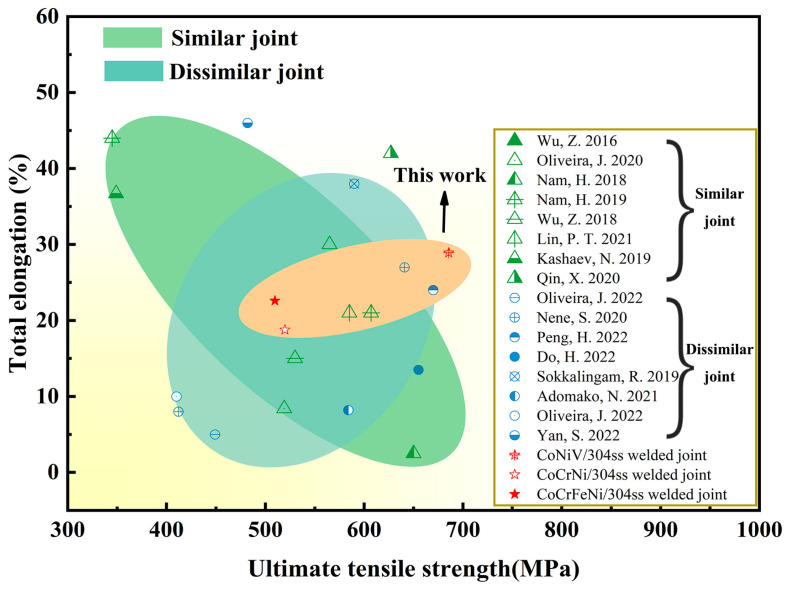
Statistics of mechanical properties of similar or dissimilar welded MCAs [12,13,14,15,17,24,26,36,38,39,40,41,42,43,44,45].

As mentioned above, the mechanical properties of a laser dissimilar welded joint between MCA and 304ss were closely related to the species of MCA, among which the CoNiV/304ss joint possesses the optimal combination of strength and ductility, i.e., YS of 338 MPa, UTS of 686 MPa, and TEL of 28.9%. Furthermore, the mechanical properties of welded joints estimated in our paper were compared to those reported results about similar and dissimilar welding of MCAs, as shown in Figure 10. It can be found that the mechanical properties of similar welded MCA joints occupied a much wide range from the low strength-high ductility combination to the high strength-low ductility combination. As a comparison, the mechanical properties of dissimilar welded MCA joints possessed relatively lower ductility combined with analogous strength. It is clear that the CoNiV/304ss welded joint shows an excellent strength-ductility synergy at room temperature, which indicates the great potential of multicomponent alloy/304ss welded joints for future industrial applications.

## 4. Conclusions

In this paper, three different multicomponent alloys (CoCrFeNi, CoCrNi, and CoNiV) were respectively applied to dissimilar laser welds with 304 stainless steel. The microstructural characteristics and corresponding mechanical properties of dissimilar laser welded joints were compared in detail to determine the optimized MCA/304ss welded joint with a high strength-ductility synergy. All of the conclusions are shown as follows:

1. All of the dissimilar laser welded MCA/304ss possessed defect-free joints accompanied by a fusion zone consisting of fcc single-phase. Concurrently, homogeneous element distribution was obtained throughout the fusion zone with a narrow element gradient existing in the vicinity of the fusion zone boundary.
2. The grain of the fusion zone mainly displayed equiaxed morphology on the side of MCA, while a coarse columnar grain was obtained on the side of 304ss. In particular, the columnar grains of the fusion zone on the side of 304ss disclosed preferential <001> growth direction in the CoCrFeNi/304ss and CoCrNi/304ss welded joints.
3. The dissimilar laser welded joints were all fractured in the fusion zone due to the drastic loss of strength in the fusion zone possessing coarsened grain. It is worth noting that the lamellar structure that merged by dimples was observed in the fracture surface of the CoNiV/304ss joint and can be associated with the existence of a V-enriched region that deformed at the latter part of the tensile process.
4. The optimal mechanical properties were obtained by laser welding the CoNiV alloy to 304ss, which presented YS of 338 MPa, UTS of 686 MPa, and TEL of 28.9%. This high strength-ductility synergy prevailed the distinct potential of the laser welded CoNiV/304ss joint to act as an excellent candidate for industrial structure.

## Figures and Tables

**Table 1:** Chemical composition and grain size of the base material.

Base Metal		Chemical Composition (at.%)						Grain Size (μm)
	Fe	Ni	Co	Cr	V	C	Bal.	
304ss	69.2	7.4	-	18.9	-	0.36	4.14	16.2
CoCrFeNi	25	25	25	25	-	-	-	14.2
CoCrNi	-	33.3	33.3	33.3	-	-	-	15.7
CoNiV	-	33.3	33.3	-	33.3	-	-	12.3

**Table 2:** Different welding base material and welded joint-hardness distribution.

	Base Material				Fusion Zone of Welding Joint		
	304ss	CoCrFeNi	CoCrNi	CoNiV	CoCrFeNi/304ss	CoCrNi/304ss	CoNiV/304ss
HV	197	166	179	353	152	158	203

**Table 3:** Mechanical properties of base materials and as-welded samples.

Sample	YS (MPa)	UTS (MPa)	TEL (%)
304ss	407	803	48.4
CoCrFeNi	315	690	43.0
CoCrNi	372	821	45.9
CoNiV	906	1279	33.2
CoCrFeNi/304ss joint	237	510	22.6
CoCrNi/304ss joint	252	520	18.8
CoNiV/304ss joint	338	686	28.9

**Table 4:** EDS results of representative positions are indicated in Figure 9f.

Position.	Chemical Composition (at.%)				
	Fe	Ni	Co	Cr	V
1	7.16	27.23	29.58	2.01	34.03
2	9.14	25.92	28.01	2.75	34.18
3	2.69	24.02	25.51	0.99	46.80
4	15.98	26.41	25.38	4.47	27.76
5	20.57	23.83	22.86	6.1	26.64

## Data Availability

The data presented in this study are available on request from the corresponding author.
